# Pathological microcircuits initiate epileptiform events in patient hippocampal slices

**DOI:** 10.1101/2024.11.13.623525

**Published:** 2024-11-14

**Authors:** Matthew A.T. Elliott, John P. Andrews, Tjitse van der Molen, Jinghui Geng, Alex Spaeth, Kateryna Voituik, Cordero Core, Thomas Gillespie, Ari Sinervo, David F. Parks, Ash Robbins, Daniel Solís, Edward F. Chang, Tomasz Jan Nowakowski, Mircea Teodorescu, David Haussler, Tal Sharf

**Affiliations:** 1. Department of Biomolecular Engineering, University of California Santa Cruz, Santa Cruz, CA, USA; 2. UC Santa Cruz Genomics Institute, University of California Santa Cruz, Santa Cruz, CA, USA; 3. Department of Neurological Surgery, University of California San Francisco, San Francisco, CA, USA; 4. Neuroscience Research Institute, University of California Santa Barbara, Santa Barbara, CA, USA.; 5. Department of Molecular, Cellular and Developmental Biology, University of California Santa Barbara, Santa Barbara, CA, USA; 6. Department of Electrical and Computer Engineering, University of California Santa Cruz, Santa Cruz, CA, USA; 7. Weill Institute of Neurosciences, University of California San Francisco, San Francisco, CA, USA; 8. Department of Psychiatry and Behavioral Sciences, University of California San Francisco, San Francisco, CA, USA; 9. The Eli and Edythe Broad Center of Regenerative Medicine and Stem Cell Research, University of California San Francisco, San Francisco, CA, USA; 10. Scientific Software Engineering Center, eScience Institute, University of Washington, Seattle, WA USA

## Abstract

How seizures begin at the level of microscopic neural circuits remains unknown. High-density CMOS microelectrode arrays provide a new avenue for investigating neuronal network activity, with unprecedented spatial and temporal resolution. We use high-density CMOS-based microelectrode arrays to probe the network activity of human hippocampal brain slices from six patients with mesial temporal lobe epilepsy in the presence of hyperactivity promoting media. Two slices from the dentate gyrus exhibited epileptiform activity in the presence of low magnesium media with kainic acid. Both slices displayed an electrophysiological phenotype consistent with a reciprocally connected circuit, suggesting a recurrent feedback loop is a key driver of epileptiform onset. Larger prospective studies are needed, but these findings have the potential to elucidate the network signals underlying the initiation of seizure behavior.

Mesial temporal lobe epilepsy (MTLE) is the most common form of drug refractory epilepsy in adults^1^. The cause of MTLE is an enigma, despite decades of research on hippocampal slices from both non-human and human brains^2,3^. For over 50 years numerous hypotheses of what may cause seizure events have been proposed and debated, with findings primarily derived from immunohistochemistry techniques^4,5^, which serve as terminal endpoints that lack functional context.

One cellular-based hypothesis of epileptogenesis points to mossy fiber sprouting in the dentate gyrus as a possible structural abnormality facilitating seizure activity (Fig. 1a). The mossy fiber hypothesis focuses on axonal outgrowths in granule cells forming new local connections in the dentate gyrus^6,7^. This increase in connectivity may establish a pathological feedback loop with self-perpetuating hyperexcitation^8^. While mossy fiber sprouting is often documented in sclerotic hippocampal specimens, its causative role in hyperexcitation is a topic of ongoing debate^9,10^.

A limitation of prior work is the level of spatial and temporal resolution of the microcircuits implicated in disease^11^. The recent advent of high-density CMOS-based microelectrode arrays (HD-MEAs) allows researchers to use densely configured recording electrodes tiled across thousands of sites to record hundreds of networked neurons across mammalian brain slices^12^. This technology is now enabling clinicians to map neural dynamics from pathological human brain tissue at spatiotemporal scales previously inaccessible^13^.

In this study, we perform a retrospective analysis of circuit dynamics from the first experiment to collect HD-MEA recordings of slices from patients suffering from MTLE^13^. Resected hippocampal tissue was sliced to 300 *μ*m, incubated, and then plated onto an HD-MEA (Fig. 1b). We analyze six slices from three different subregions of the hippocampus: the inner apex of the dentate gyrus, the outer blade of the dentate gyrus, and CA1 (Supplementary Table 1)^14^. Two slices with electrodes covering the outer blade exhibited seizure-like behavior after the administration of kainic acid. In these two slices from separate patients, we observe a strikingly similar pattern of neuronal firing and local field potential (LFP) oscillations in the theta band (4–8 Hz) that propagate as coherent waves during the onset of epileptiform bursting activity. The onset of these network activity patterns, which are cyclic in nature, may represent a recurrent, pathologic circuit initiating epileptiform behavior in the dentate gyrus.

We used the high resolution of the HD-MEA to build anatomical maps of neural behavior within subregions of the hippocampus. These maps are based on histochemical stains. Prior to recording, slices were transduced with an adenoviral vector carrying a channelrhodopsin (HcKCR1) and eYFP fusion protein on a CAMK2A promoter (AAV9-CAMK2A-HcKCR1-eYFP). Slice immunohistochemistry utilized anti-NeuN (magenta) and anti-GFP (cyan) antibodies, demonstrating dense staining of CAMK2A-expressing neurons clustered in the granule cell layer of the dentate gyrus (Fig. 1c). This is consistent with single nucleus sequencing data documenting high expression of CAMK2A in granule cells compared to other hippocampal neurons^15^. While the electrophysiologic data described here was gathered from experiments that utilized optogenetic activation of the channelrhodopsin, for the purposes of these analyses, optogenetic data is not analyzed (see Discussion).

To visualize the high-density electrophysiologic data, we project the locations of neurons and electrodes onto a 2D map. The locations of recording electrodes and neural units discerned through spike sorting were overlaid on the histology images by best approximation for all six slices (Fig. 1d, Supplementary Fig. 2). The HD-MEA provides resolution at the level of an individual neuron’s spatial footprint, allowing for high-resolution spatial plots of neural behavior (Fig. 1e–f).

We began our analysis by assessing whether epileptiform behavior was present in the data. After administering kainic acid to slice S1, synchronized rhythmic bursting activity was observed (Fig. 1g–h). Each cluster of rhythmic bursting activity (Fig. 1g) is defined as a superburst (as termed by Wagenaar)^16^. Fig. 1g contains five superbursts. Each superburst has 6–8 bursts (Fig. 1h). A spectrogram from S1 displays local field potentials power during the first superburst (Fig. 1i). Spectral characteristics consistent with seizure-like events are present. These include a defined power increase in theta band activity, coherence between theta activity and bursting behavior, and a large upwelling in the delta frequency at epileptiform onset^17–19^. These characteristics were also present across different electrode sites where the LFP traveled as a coherent wave (Supplementary Fig. 3). Epileptiform behavior was also present when kainic acid was administered to slice S2 (Supplementary Fig. 5).

To understand what might initiate seizure-like behavior, we investigated theta wave behavior at the onset of rhythmic bursting activity. To present the high density electrophysiologic data while preserving its spatial dimension as well as its time dimension, we projected the theta voltages from approximately 1000 electrodes onto the slice histology to label neurons (Fig. 1j). In these plots red represents positive voltage and blue represents negative voltage. The changing dynamics of the theta wave propagations are best observed by watching Video 1 (https://youtu.be/wj1TvUE-KZI).

We observed unique theta propagations at the onset of seizure-like behavior that transition between distinct, orthogonal modes. The first superburst following kainic acid administration on slice S1 (Fig. 1i) was divided into three stages, baseline (prior to burst activity), initiation (the first burst), and seizure (subsequent bursts)^20,21^, based on theta wave propagations (Fig. 1j, Video 1). At baseline, there is no discernable pattern of theta propagations. The initiation stage occurs at the onset of seizure-like behavior^22^. During the initiation stage, theta propagations form a standing wave that oscillates across the length of the granule cell layer (Supplementary Fig. 4d). These theta oscillations across the long axis of the granule cell layer observed in the initiation stage (Fig. 1j) are unique to the first burst of the recording (Video 1–4). In the seizure stage theta propagations form a rolling wave, moving orthogonally to the initiation stage, along the short axis of the granule cell layer from the hilar aspect to the outer aspect (Supplementary Fig. 4e). The same stages of baseline, initiation, and seizure were observed when kainic acid was administered to slice S2 (Supplementary Fig. 5).

We checked that observed theta propagations represent the primary direction of oscillatory movement within each stage and are not artifacts. For each stage of the recording, electrodes were clustered based on their average time delay between theta propagations (Supplementary Fig. 4g). With this technique, clusterings present the directionality of the dominant wavefront for each stage. Spatial maps of the electrode clusterings replicated the behavior seen in theta wave propagations (Supplementary Fig. 4h). The slices with no epileptiform activity (S3-S6) did not contain strong theta wave propagation patterns (Supplementary Fig. 6).

We next considered if the unique theta propagations observed at the initiation stage might reflect some distinct pattern of neural firing activity. We constructed spatial plots of changes in the unit firing rate and compared them to the timing of theta propagations. We found that theta wave activity during the initiation stage of S1 is temporally aligned with oscillations in spiking activity, suggesting some form of recurrent feedback at epileptiform onset^8^. Neural activity from the first burst of S1 was divided into three sub-bursts (Fig. 2a). The peaks and troughs of sub-bursts aligned with when the two modes in theta activity were maximally distinct from each other. Calculating the change in unit firing from sub-burst to sub-burst produced heat maps with similar oscillations to those seen in theta propagations. Consistent results suggest there is also a recurrent feedback loop at initiation in the second slice (S2) with epileptiform activity (Supplementary Fig. 5f). A different pattern of coherence between theta waves and spiking behavior occurred during the seizure stage (Supplementary Fig. 7, Video 3–4).

After analyzing neural dynamics during epileptiform onset, we next considered if these dynamics may be part of a larger pattern present across the entire length of the recording. Such a pattern might suggest an underlying mechanism related to the behavior observed in the initiation stage. Phase locking is a common tool for measuring the synchronization between neural spiking behavior and theta wave rhythms^23^. Phase locking analysis across the entire recording provided results consistent with those seen in the initiation stage (Fig. 2b). Significantly phase locked units were determined by considering the correspondence between their spike times and theta wave phase (Rayleigh p-value < 0.05). A heatmap of the difference in phase angles between significant units produced a bimodal plot similar to those previously shown. Clustering based on the two modes of significant units yielded two groups with significantly different phase angles (Kuiper’s p-value = 0.01). When evaluating the phase locking dynamics across all slices, we observed considerably more phase locked units in the two slices with epileptiform activity, with phase locking particularly high during seizure-like events (Supplementary Fig. 8, Supplementary Table 2).

Spiking behavior across the entire recording recapitulates the spatial dynamics observed in phase locking (Fig. 2c). Two groupings of neurons were created by performing hierarchical clustering on the spike time tiling coefficient (STTC) matrix, a more robust analog of the correlation matrix^24^. A spatial map of the neural clusters divides the granule cell layer into the same two modes seen in previous plots. Similar results were observed in the second epileptiform slice (S2) (Supplementary Fig. 5h). A second clustering method based on the eigendecomposition of the STTC matrix reproduced these results (Supplementary Fig. 9).

We noticed a marked resemblance in the spatial plots from the initiation stage (Fig 2a) when compared to the spatial plots from our whole recording analysis (Fig 2b–c). This led us to ask if the high neural resolution of the HD-MEA could be used to unveil a structural quality of the slice that might explain recurrent feedback. Furthermore, we wanted to check that the initiation stage was not simply a byproduct of seizure-like activity. In a sophisticated in silico simulation of the dentate gyrus (Supplementary Fig. 13), we found that increasing interconnectivity between granule cells produced seizure events. This led us to consider if constructing anatomical diagrams of neural microcircuits might elucidate interconnectivity within our slices.

We created neural circuit diagrams that display the directions that neural spiking signals propagate across the tissue as a vector plot of arrows. Each arrow is a significant circuit connection, representing the primary direction that spikes emanate from a given neuron, with color denoting the angle of the arrow (Fig. 2d). Connections are rigorously derived from analyzing spike time latencies between neural pairs^25^ (see Methods). The circuit diagram for slice S1 (Fig. 2e) displays two clusters of arrows (green and red), consistent with the bimodal behavior seen in previous plots. Each cluster is propagating spike signals toward the other, suggesting a high degree of neural interconnectivity. The connection angle histogram (bottom-right of Fig. 2e) plots the total number of spike events by connection angle. It displays a bipolar graph with spike propagations traveling 180° along a single axis. Such bimodal interconnectivity would explain the recurrent feedback loop observed at seizure-like initiation.

We can compare the circuit diagrams and the connection angle histograms from all eight recordings to check if there is a unique microcircuit inherent to the epileptiform slices (Supplementary Fig. 10). The second slice with epileptiform activity (S2) also had a bimodal circuit diagram, with spikes propagating toward each other, and a bipolar connection angle histogram. This, again, is consistent with the recurrent feedback observed at seizure-like initiation for S2. For both S1 and S2, the non-epileptiform recordings prior to administering kainic acid had the same circuit diagram and connection angle histogram as during epileptiform activity. This suggests that the bimodal interconnected circuit of S1 and S2 is a structural quality of the slices and not a by-product of seizure-like activity.

The non-epileptiform slices did not display the bimodal circuit diagram or bipolar connection angle histogram seen in the epileptiform slices (Supplementary Fig. 10). Both slices from the inner apex of the dentate gyrus (S3 and S4) had a unimodal connection angle histogram, with spike signals propagating toward the inner blade. Notably, slice S4 was administered kainic acid, but did not exhibit epileptiform behavior. The slices from CA1 (S5 and S6) had evenly distributed circuit diagrams and a bimodal, but not bipolar, connection angle histogram, with spike signals propagating along the perforant pathway. In the case of all recordings, slices from the same subregion followed the same circuit behavior. A statistical test was performed to check if the differences we observed in circuit diagrams were due to bias in the geometric layout of neurons (Supplementary Fig. 12). If that were the case, the test’s p-values would be similar for all slices, however, we found the epileptiform slices to be orders of magnitude more significant (Supplementary Table 3).

In summary, using the high resolution of an HD-MEA, we observed a striking relationship between the spatial dynamics of epileptiform onset and circuit level behavior across the entire recording. Two hippocampal slices from different epilepsy patients display a bimodal recurrent circuit in the same subregion of the dentate gyrus, the outer blade. Furthermore, recurrent oscillations in theta waves and neural firing activity are observed between these two modes at the initiation of epileptiform behavior. These results provide electrophysiological evidence of pathological feedback leading to hyperexcitation in the dentate gyrus. This may be the locus of initiation for seizure events.

This study has limitations. The availability of samples was scarce due to this being a retrospective analysis of the first experiment to date to use HD-CMOS technology on human brain slices^13^. Seizure-like slices had to be compared against still unhealthy, non-seizure-like slices excised from epilepsy patients. There are no viable controls even from animal models, because current HD-MEA technology requires hippocampal slices from large mammals to observe subregional activity. Confounding factors are introduced by the original experiment. Notably, organotypic slices, instead of acute, were used and optogenetic inhibition was performed on the latter portion of the kainic acid recordings (after epileptiform initiation).

In conclusion, recent advancements in HD-MEA’s allow for the study of neural dynamics at microscopic resolution, offering a new approach to validate proposed models of circuit behavior. When applied to MTLE, our finding of feedback-driven hyperexcitation in the dentate gyrus is consistent with the mossy fiber sprouting hypothesis of epileptogenesis. By integrating these computational methods with novel biological approaches for elucidating microscopic neural circuits^26^, dedicated prospective studies have the potential to both localize and verify the neurological mechanisms of epilepsy. A more localized understanding of MTLE may lead to less invasive surgical procedures^27^, and perhaps one day, new pharmacological treatments.

## Methods

### Tissue preparation

Samples were collected from patients undergoing temporal lobectomy with hippocampectomy for refractory epilepsy. We obtained signed patient consent and approval from the University of California-San Francisco Institutional Review Board. The tissue was sliced into 300μM sections. Slices were plated on cell-culture inserts at the air-liquid interface, transduced with a CAMK2A promoter via an adeno-associated virus, and then incubated for 4–8 days prior to recording. On the day of the recording, slices were incubated for one hour and then plated on HD-MEAs with minimal culture media. For slices with Kainic acid experiments (S1, S2, and S4), 100nM kainic acid was dripped directly onto the slice. For further details see the original study^1^.

### Experimental design, reproducibility, and inclusion/exclusion criteria

The sample size was maximized based on the availability of human brain tissue. Experiments were run in the order that tissue became available. No randomization was performed. Data collection and analysis were not performed blind to the conditions of the experiments. All the slices that were analyzed were from adult patients with refractory epilepsy.

We required higher levels of neural activity than what was necessary for the original experiment. We excluded any slice with less than 50 neurons after spike sorting. Of the 12 slices from the original study, six were analyzed. For transparency, results from all 6 slices are present in our analysis. See Supplementary Fig. 1,2,10 and Supplementary Tables 1–3. The main figures display results for the primary slice with epileptiform activity, S1. Corresponding results from the second slice with epileptiform activity, S2, are in Supplementary Fig. 5. Results for the four non-epileptiform slices (S3–6) are in Supplementary Fig. 6.

#### Immunohistochemistry:

**Fig. 1 |:**
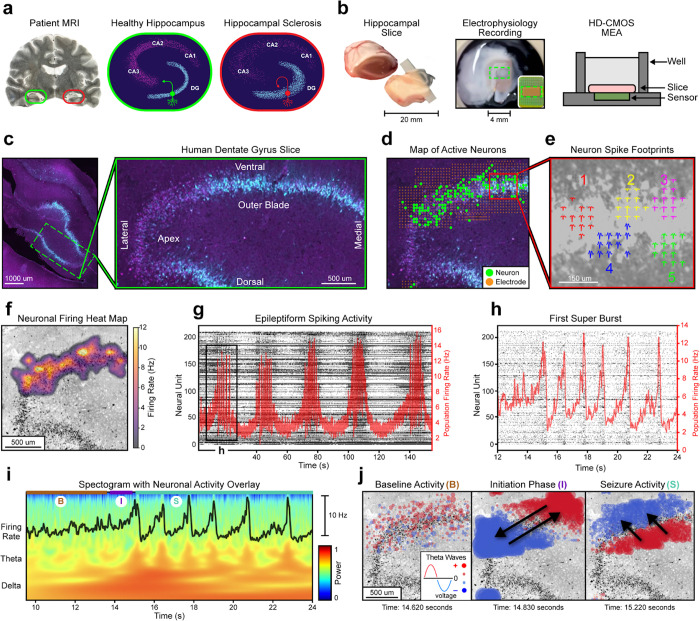
Spatially mapping epileptiform dynamics within the human dentate gyrus. **a,** Left: Patient MRI highlighting hippocampal sclerosis (red) compared to healthy tissue (green). Middle: Diagram of a coronal section from a healthy hippocampal slice. Primary regions are labeled (DG, CA1–3), and a depiction of a standard granule cell is displayed (green). Right: Diagram of sclerotic tissue. Exhibited pathologies include cellular loss, granule cell dispersion, and mossy fiber sprouting (red neuron). **b,** Resected patient hippocampal tissue is sliced to 300*μ*m and placed on an HD-CMOS microelectrode array. A green rectangle depicts the region where electrophysiology data is recorded. **c,** NeuN (magenta) and eYFP (cyan) immunohistochemistry of a hippocampal slice transduced with AAV9-CAMK2A-HcKCR1-eYFP, with cyan representing CAMK2A expressing cell clustered in the granule cell layer of the dentate gyrus. Right: Magnified image of dentate gyrus, with subregions labeled. **d,** Spatial map of recording electrodes and neural units from the slice in **c**, discerned through spike sorting. **e,** Spatial footprints of five neurons action potentials from inset in **d**. For each neuron, an averaged 3ms action potential is displayed on top of its recording site. **f,** Heatmap visualization of neuronal firing rates collected across the duration of the recording from **d**. **g,** A raster plot of spike events shown as black dots (left axis) with the population average firing rate shown in red (right axis). Neural activity is from the first 150 seconds of epileptiform activity for the slice in **d**. Five superbursts are displayed. **h,** A zoomed in view of the first superburst from **g**. **i,** A spectrogram from the first superburst highlighting delta and theta band activity with the firing rate overlayed on top. Based on theta wave behavior, the superburst is divided into three sections, baseline (B), initiation (I), and seizure (S) stage. **j,** Spatial plots of theta waves during baseline, initiation, and seizure stage. Red signifies a positive voltage, and blue signifies a negative voltage. Black arrows display the direction of wavefront propagations across time. See Video 1 (https://youtu.be/wj1TvUE-KZI).

For the histology images shown in Fig. 1c–d and Supplementary Fig. 1, the following antibodies were used for immunohistochemistry. Slices were infected with the AAV9-CAMK2A-HcKCR1-eYF adeno-associated viral vector prior to staining.

NeuN: guinea pig anti-NeuN, Millipore, ABN90, dilution 1:1000, lot#4077530

eYFP: Chicken anti-GFP antibody, Aves, GFP-1020, dilution 1:1000, lot# GFP3717982

### Data acquisition and spike sorting

Extracellular field potentials were sampled at 20kHz from up to 1,024 electrodes using an HD-CMOS microelectrode array (MaxOne, Maxwell Biosystems, Zurich, Switzerland)^2^. The array contains 26,400 recording electrodes with a diameter of 7.5μm at a center-to-center distance of 17.5 μm. At the beginning of the experiment, an activity scan assay was performed across all electrodes. Approximately 1,000 recording electrodes were manually selected based on the most active regions found in the scan. After the experiment, raw activity data was saved to an HDF5 file on local memory.

Raw extracellular recordings were bandpass filtered between 300–6000Hz and then spike sorted in Kilosort2^3^ to extract single neural unit locations and activity. Sorting was performed on the Pacific Research Platform computing cluster^4^. Kilosort2’s results were manually curated using Phy GUI^5^ by experienced researchers who took into consideration each unit’s spike waveform, correlogram, and interspike interval violations.

### Spatially mapping electrodes and neurons

Fig. 1d and Supplementary Fig. 2 display the spatial locations of recording electrodes and neural units mapped to histology images. After a slice was plated onto the microelectrode array, an upright microscope (MS08B, Dino-Lite) photographed the tissue. We mapped neurons and electrodes by comparing the microscopy image to the histology. Specific locations of electrodes were extracted from the H5 file produced by the recording. The placement of neural units was provided by the Kilosort 2 spike sorting algorithm.

### Spike rasters with population level firing activity

**Fig. 2 |:**
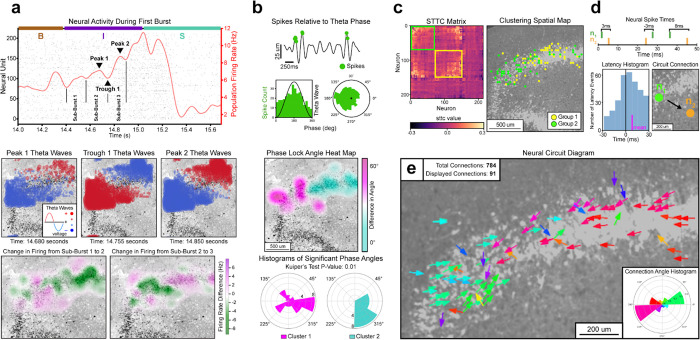
Recurrent feedback within a pathological microcircuit. **a,** Top: Neural activity from the first burst of the recording with baseline, initiation, and seizure phases labeled by B, I, and S, respectively. In the initiation phase, sub-bursts, peaks, and troughs are labeled. Middle: Spatial plot of theta wave activity taken at the time points of the labeled peaks and troughs. Bottom: Heatmap of the change in firing from sub-burst to sub-burst. During initiation, firing activity oscillates between the two modes observed during theta wave activity. **b,** Top: Example of a neural unit that is significantly phase locked (Rayleigh p-value<0.05). Spike events are projected to their corresponding location on a theta wave. Two histograms, polar and nonpolar, display the frequency of spikes based on theta phase angle. Middle: Heatmap of the average difference in phase angles between all significantly phase locked units across the entire recording. Bottom: Polar histograms of neural phase angles from the two clusters in the heatmap above. Clusters have significantly different angular distributions (Kuiper’s P-value<0.01) **c,** Left: The spike time tiling coefficient matrix, with units organized based on agglomerative hierarchical clustering. Green and yellow squares indicate groupings observed from clustering. Right: Spatial plot of neurons based on their grouping from the STTC matrix. **d,** Schematic of the process for defining a circuit connection. Spike raster depicts latency times calculated from a neural spike pair (n_1_,n_2_). A histogram from the pair illustrates that the latencies are significantly different from zero (t-test p-value<1e-10, mean=4.7ms), leading to an arrow being drawn between (n_1_,n_2_). **e,** A neural circuit diagram displaying 91 of the 784 significant circuit connections. Connections are colored based on the angle that spikes propagate. The connection angle histogram (bottom-right) displays the angular frequencies from all spike propagation events. The microcircuit is bimodal, containing a green and red grouping of connections. The connections between these groupings propagate toward each other, suggesting pathological recurrent behavior in the circuit.

Fig. 1g–h and Fig. 2a contain neural spike rasters with the population level firing rate overlayed on top. The population firing rate is calculated by, first, summing the total number of spikes in each millisecond bin. A moving average of the spike counts is created by applying a 1D Gaussian filter to the bins, with a standard deviation between 10–20ms. Each 1ms bin of spike counts is divided by the total number of neurons and multiplied by 1000 in order to correspond to the standard formula for firing rate.


$$Population\;Firing\;Rate\;(Hz)=\frac{\text{Number}\;\text{of}\;\text{Spikes}}{\text{Number}\;\text{of}\;\text{Neurons}\;\times \;\text{Time}\;\text{(seconds)}}$$

### Neuronal Firing Activity Heatmaps

A spatial heatmap of firing activity is displayed in Fig. 1f, illustrating the average neuronal firing rate (in Hz) across the granule cell layer. First, the average firing rate for each neuron was calculated by dividing the total number of spikes by the recording duration. Neurons were spatially mapped into a grid of 900 squares, each measuring approximately 58.3 μm × 58.3 μm. The average firing rate for each square was calculated based on the neurons it contained. A 2D Gaussian filter was applied to the grid in order to smooth the spatial distribution of firing rates. The filter replaces each point with a weighted average of its neighboring points. These weights are determined by a 2D Gaussian distribution, with a standard deviation of 37.9 μm. Fig. 2f and Supplementary Fig. 5f display heatmaps of the difference in firing activity between neuronal sub-bursts using a method analogous to the one described above.

### Neuron Spatial footprints

Fig. 1e displays individual neurons’ spatial footprints. Kilosort 2 saves the putative footprints for each neural unit found through spike sorting. Representative electrodes are detected for each neural unit by averaging the amplitudes across spikes and choosing the 12 channels with maximum amplitude. The waveforms displayed are an average of the action potentials that occurred for that electrode.

### Spectrogram

Fig. 1i and Supplementary Fig. 3 display spectrograms. The spectral analysis shows the signal strength of different subbands in the local field potential of a single electrode. We first filter the raw voltage signal with a bandpass filter between 0.1–100 Hz. Signals are downsampled from 20 kHz to 1 kHz. A second bandpass filter is then applied to extract the subband frequencies of delta (0.5 – 4 Hz), theta (4 – 8 Hz), alpha (8 – 13 Hz), beta (13 – 30 Hz) and gamma (30 – 50 Hz) waves. To plot the spectrogram, we run a continuous wavelet transform on the local field potential data. This transform is performed using the complex Morlet wavelet (‘cmor1–1’ in pycwt), which computes wavelet coefficients and corresponding frequencies. The power is computed as the magnitude squared of the wavelet coefficients and then smoothed using a Gaussian filter (sigma=2).

### Theta wave activity plots and videos

Theta wave activity plots provide a spatial map of the theta wave values from all, roughly, 1000 electrodes at a single cross-section in time (Fig. 1j and Supplementary Fig. 4–5). A standard neuroscience protocol was used to calculate the theta wave values for each electrode. A fourth order Butterworth bandpass filter selecting frequencies between 4–8Hz was applied to the raw voltage data from each electrode. For visualization in the spatial plots, resulting theta waves are normalized by electrode, with values between [−1, 1]. The theta value from each electrode is represented by a circle, centered at the spatial location of the electrode. The color of the circle is red if the voltage of the electrode’s theta wave value is positive, and blue if it is negative. The size of the circle scales with the absolute amplitude of the voltage, making circles with a higher magnitude larger.

Every theta wave activity plot has a corresponding video, which provides a clearer understanding of how theta wave propagations evolve through time (Supplementary Videos 1–4). Videos of theta wave activity move at a pace 10–20 times slower than real time. The frames in the video change at a 5ms time interval. The video displays theta activity on the left and neural firing activity on the right. Having these plots side-by-side is useful for understanding the coherence between theta activity and population level firing activity (see Video 3, Supplementary Fig. 7).

### Electrode spatial clustering algorithm

For slices with epileptiform activity (S1 and S2), theta propagations observed in the baseline, initiation, and seizure phases (Video 1) were verified using a spatial clustering algorithm (Supplementary Fig. 4g and 5e). The clusterings resemble the theta activity seen for the corresponding phase. Electrodes were clustered based on the average time delay (lag time) between theta waves as they propagated across the electrodes. Cross-correlation analysis was performed between all pairs of electrodes (scipy function: correlate). The lag time (ms) that maximized the absolute correlation between a pair of electrodes was selected as the pair’s time delay. Only lag times between [−40,40] ms were considered. A square matrix is created using the lag times from all electrode pairs (Supplementary Fig. 4f). K-means clustering is performed on the lag times matrix using an N of two clusters. The spatial clusterings of the electrodes are then observed by plotting electrodes by location (Supplementary Fig 4g).

### Phase locking neurons to theta wave activity

Phase locking analysis was performed in Fig. 2b, Supplementary Fig. 8, and Supplementary Table 2^6^. First, the phase angles of the LFP filtered in the theta frequency band (4–8Hz) were obtained by a standard Hilbert transformation on the time series and subsequently calculating the angle between the real and imaginary components. For each spike sorted unit, the upper envelope of the theta filtered LFP was also calculated. For all spikes that occurred while this upper envelope was above the RMS of the whole time series, the theta phase angle of the spikes were stored. The Rayleigh criterion was used to test the non-uniformity of the phase angles over all the selected spikes (0°, 360°). Spikes were considered to be phase-locked to theta if they passed the Rayleigh criteria test for non-uniformity (p < 0.05). This was done separately for seizure and non-seizure phases in the recording (Supplementary Fig. 8). For each significantly phase-locked unit, the circular mean was computed over all selected spikes to obtain the average phase locked angle. The Rayleigh criteria test for non-uniformity is the polar analog to the one-sample T-test. Its R-statistic (analog to t-statistic) is calculated as follows:

$$R=\sqrt{{\left(\sum_{i=1}^{N}\;cos\;\sigma_{i}\right)}^{2}{\left(\sum_{i=1}^{N}\;sin\;\sigma_{i}\right)}^{2}}\;\begin{array}{l} N=\text{total}\;\text{samples}\\ \sigma_{i}=\text{angle}\;\text{of}\;i\text{th}\;\text{neuron} \end{array}$$

### Methodology for heatmap of phase locked angles

Heatmaps presenting the spatial difference in phase locked angles across the tissue were presented for slices S1 and S2 (Fig. 2b and Supplementary Fig. 5g). Heatmaps were constructed using only neurons that were significantly phase locked (Rayleigh p-value<0.05). The polar mean was used to find the primary phase angle each neuron was phase locked to. A polar histogram of all significant phase angles was constructed to determine the most frequently occurring angular direction (polar mode). The absolute angular difference from the polar mode (L1 norm) was calculated. These differences were used to construct a spatial heatmap of polar angles.

Given the angular values of each neuron, the methodology for constructing the heatmap is similar to the heatmaps for firing activity described above. Neurons’ angular values were mapped to a grid of squares, with the value of each square calculated based on the average of the neurons it contained. Then a 2D Gaussian filter was used to construct a smoothed spatial distribution, using a standard deviation of 70μm. The polar histograms next to the heatmap display angular frequencies corresponding to the two clusters observed from the heatmap. They are created by partitioning the phase locked neurons based on their angular difference and then plotting the resulting clusters. The Kuiper test was used to compare the angular distributions between the two clusters.

### Methodology for STTC hierarchical clustering of neurons

For both epileptiform recordings (S1 and S2), we provide spatial plots of neural clusters (Fig. 2c and Supplementary Fig. 5h). To create the clusters, a standard agglomerative hierarchical clustering algorithm was implemented on the spike time tiling coefficient (STTC) matrix. Because agglomerative clustering is sensitive to outliers, the STTC matrix’s maximum value was thresholded to 0.3 (roughly the 98th percentile). Clustering was done using the scipy.cluster.hierarchy package. The Euclidean pairwise distances between all pairs in the STTC matrix were calculated. Agglomerative hierarchical clustering was done on the pairwise distance to construct a linkage matrix corresponding to a dendrogram. The resulting hierarchical clustering tree was reordered to reflect the optimal leaf arrangement. The STTC matrix was reordered based on the optimal leaf ordering (Fig. 2c). Two clusters were observed in the resulting matrix. The neurons from these clusters were plotted spatially.

### Definition of circuit diagram connection

In the circuit diagram figures (Fig. 2d and Supplementary Fig. 10), an individual arrow is defined as a circuit connection. A circuit connection is a spike propagation vector between two neurons, where the base of the arrow is the location of the neuron from which the spike emanates and the angle of the arrow points toward the neuron that spikes afterward. Arrows are drawn at a constant fixed length, usually shorter than the distance between neural pairs. This is done to declutter the circuit diagram.

Circuit connections are constructed by considering the latency events between every pair of neurons in the dataset^7^. Figure 2d illustrates the methodology used to construct circuit connections. Given a pair of neurons (n_1_,n_2_), for every spike that occurs in n_1_, we find the nearest occurring spike from n_2_, using the absolute difference in time as our metric. We construct a latency distribution by considering events that occur within a window of [−30ms,30ms]. A two-tailed t-test is performed on the latency distribution to determine if it is significantly different from zero (p value < 0.05). A circuit connection is created for all significant pairs. Of the 45,000 pairs of neurons in slice S1, 784 pairs were classified as circuit connections.

A standardized protocol constructed circuit diagrams for all recordings (Supplementary Fig. 10). To reduce computation time, only neural pairs with a spike time tiling coefficient value greater than 0.01 were considered. Pairs had their latency distributions calculated. Latency distributions with less than 25 latency events or with an absolute mean latency less than 1ms were disregarded. A two-tailed t-test was performed on all remaining pairs. If the pair’s p-value was significant (<0.05), it was considered a circuit connection.

### Displaying circuit connections

The neural circuit diagrams in Supplementary Fig. 10 display only a fraction of all connections. Displaying all connections would make the plot difficult to interpret. Supplementary Fig. 11 illustrates the protocol used to select a representative subsample of connections.

First, we aggregate circuit connections based on the neuron that is propagating the signal. Multiple connections emanating from a neuron are replaced with a single arrow. The polar mean (scipy: circmean) is used to aggregate the angles of all emanating connections, with connections weighted by their number of latency events. Aggregating connections sometimes displays arrows that are not representative of the original connection directions (see Extend Data Fig. 11, bottom neuron example). Connections pointing in opposite directions are averaged to form a new arrow, not representative of either original connection. Nonrepresentative connections are removed by only considering aggregated connections whose standard deviation is below 0.5. The weighted polar standard deviation is used (scipy: circstd), with weights based on latency events. The remaining connections are displayed in the circuit diagrams

### Connection angle histogram

At the bottom of every circuit diagram is an inset displaying the connection angle histogram (Supplementary Fig. 10). The connection angle histogram displays the angular frequency of spike propagations across all circuit connections. The connection histogram is calculated over all circuit connections, not just the displayed connections (see above). Also, connections are weighted based on the number of latency events that occur within that connection. Colors in the histogram correspond to connections (arrows) of the same angle.

### Statistical test of circuit geometry

A statistical test was performed to check whether or not the recurrent circuits seen in the epileptiform slices (S1 and S2) are due to a bias caused by the geometry of neuronal locations. For all recordings, the spatial layout of neurons roughly followed a line across the granule cell layer (Slice 1–4) or pyramidal cell layer (Slice 5–6) (Supplementary Fig. 2). This test checks whether or not the relative location of neurons is the determining factor in the angle of circuit connections. Supplementary Fig. 12 provides an illustrative schematic of the test. To find the relative positioning between neurons in a recording, they were first projected onto a line that goes through the center of the cell layer. The line was approximated by fitting a polynomial regression of degree two to all neurons. Neural positions were normalized with the leftmost position along the curve being 0.0 and the rightmost being 1.0. A scatter plot was created comparing the relative position of neural connections to their corresponding angle (in Radians). All connections were divided into two groups based on whether the connection angle was positive or negative. A two-tailed t-test was performed on the neural positionings of the two groups to determine to what extent they were significantly different.

If the observed circuit diagrams were due to bias in the neural geometry, the test’s p-values would be roughly similar for all slices, because relative neural positionings would be the determining factor in circuit angle. However, we found the pathological circuit recordings to be orders of magnitude more significant, verifying that the phenomena we observe is not due to bias in neural geometry (Supplementary Table 3).

### Eigendecomposition for neural spatial clustering

We perform an eigendecomposition on the STTC matrix to spatially cluster neurons for slices with epileptiform activity (S1 and S2). An illustrative schematic of the methodology and its results are displayed in Supplementary Fig. 9. First, the STTC matrix was calculated. The STTC matrix is analogous to the commonly used correlation matrix, but has been shown to perform more robustly on neural data. For our data, the STTC matrix had higher eigenvalues and a lower reconstruction error when compared to correlation. Like the correlation matrix, the STTC matrix is positive semidefinite, which means its eigendecomposition can be mathematically interpreted similarly to that of Principal Components Analysis (PCA). We plot the values from the first eigenvector spatially by coloring each neuron based on its eigenvector value. This results in a gradient that travels across the granule cell layer, similar to the bimodal clusters observed from hierarchical clustering (Figure 2c).

### Simulation of epileptiform behavior

We created a dynamical simulation of a hippocampal network as a proof of concept. Our model is a simplified version of a previously published in silico model of the human dentate gyrus which has been used to study disease progression in epilepsy^8^. Simulated connections between granule cells in the dentate gyrus were added as a variable fraction of the total cells within this model in order to probe its effect on seizure-like events. For further details, see the “Simulation” directory in the Github repository.

## Supplementary Material

Supplement 1

## Data Availability

All data used in the analysis and creation of figures is available in the GitHub repository: https://github.com/braingeneers/human_hippocampus The repository includes neural spiking data, histology images, portions of raw electrophysiological data, and plots created for figures. The complete dataset of raw electrophysiological recordings from experiments is available on the DANDI public server: https://dandiarchive.org/dandiset/001132
